# Supplementary material for: Probabilistic data integration identifies reliable gametocyte-specific proteins and transcripts in malaria parasites
Source: Sci Rep. 2018 Jan 11;8:410. doi: 10.1038/s41598-017-18840-7 (PMC5765010; doi:10.1038/s41598-017-18840-7)
Supplement: Supplementary file 1 — Supplemental Figures [file 41598_2017_18840_MOESM1_ESM.doc]

SUPPLEMENT

Probabilistic data integration identifies reliable gametocyte-specific proteins and transcripts in malaria parasites

Lisette Meerstein-Kessel[[1]](#footnote-2),[[2]](#footnote-3), Robin van der LeeError: Reference source not found^#a^, Will Stone^2^,[[3]](#footnote-4), Kjerstin Lanke1, David A Baker[[4]](#footnote-5), Pietro Alano[[5]](#footnote-6), Francesco SilvestriniError: Reference source not found, Chris J Janse[[6]](#footnote-7), Shahid M KhanError: Reference source not found, Marga van de Vegte-Bolmer1, Wouter Graumans1, Rianne Siebelink-Stoter1, Taco WA Kooij1, Matthias Marti[[7]](#footnote-8), Chris DrakeleyError: Reference source not found, Joseph J. Campo[[8]](#footnote-9), Teunis JP van DamError: Reference source not found^#b^, Robert Sauerwein^2^, Teun Bousema1^¶^, Martijn A HuynenError: Reference source not found^¶^*

**Supplementary material**


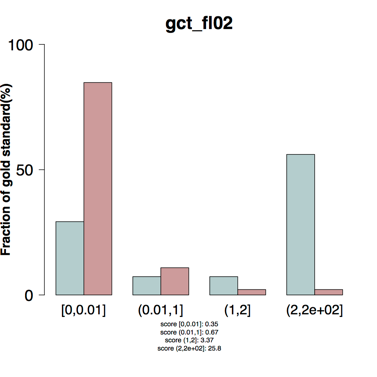

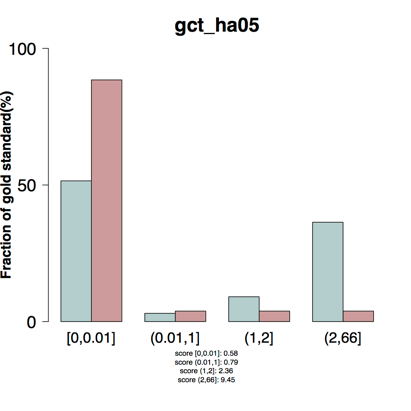

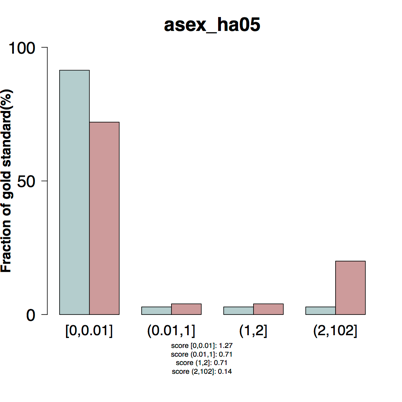

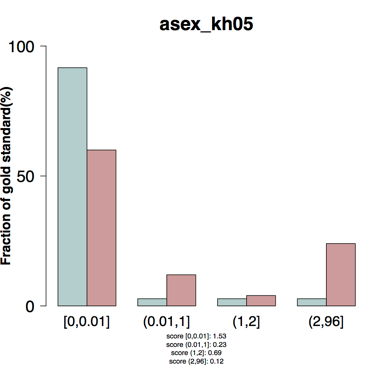

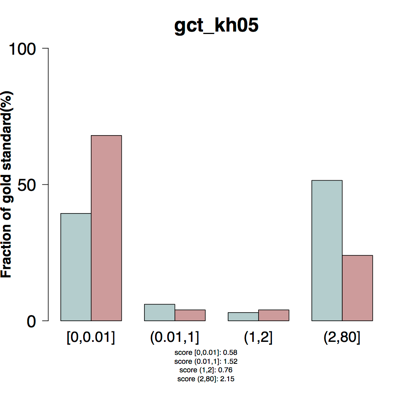


Supplementary Figure 1 (continues on next page)


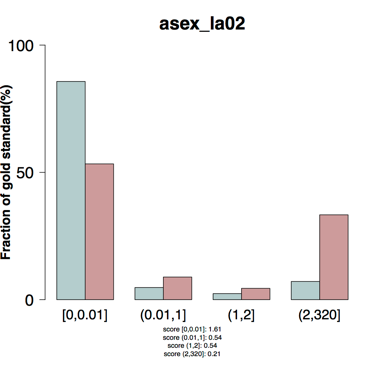

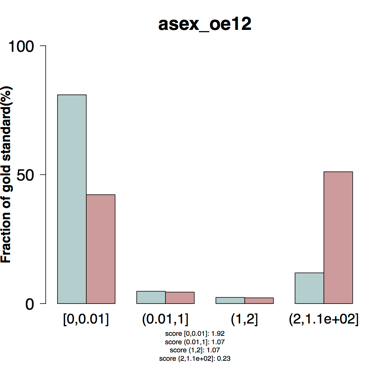


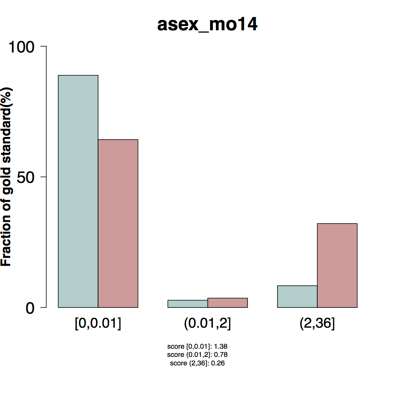

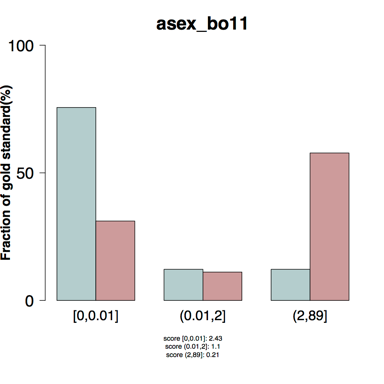

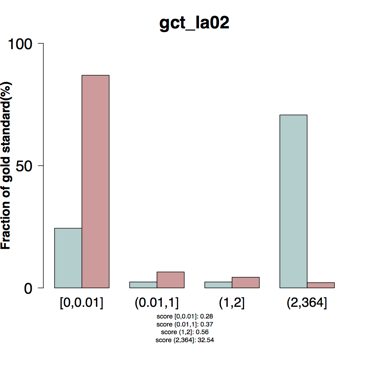

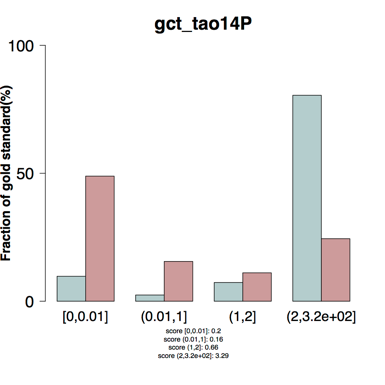


Supplementary Figure 1 (continues on next page)


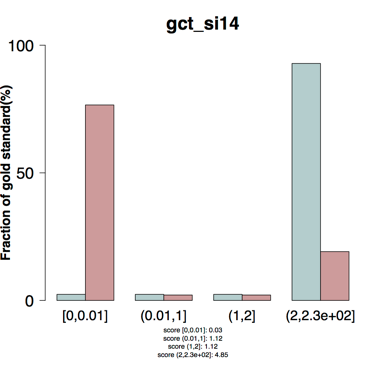

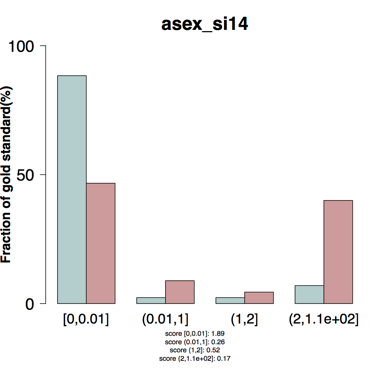

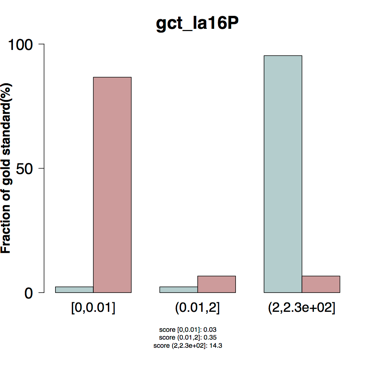

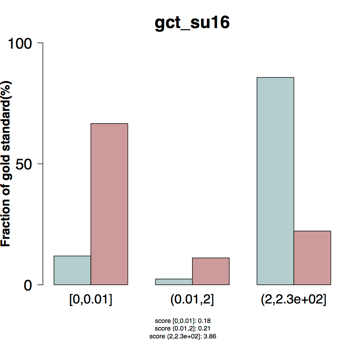


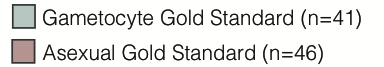

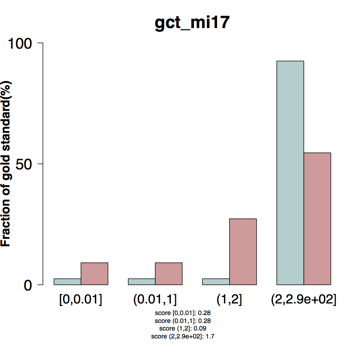


**Supplementary figure 1**. Separation of gametocyte (blue) and asexual (red) gold standard (GS) genes by proteomics studies. See Table 1 for study keys. Bins for no unique peptides [0,0.01], one unique peptide (0.01,1] two unique peptides (1,2] or more than two unique peptides (2, max] retrieved. Scores for each bin are calculated as the log2-transformed fraction of gametocyte GS genes over asexual GS genes retrieved in the respective bin.


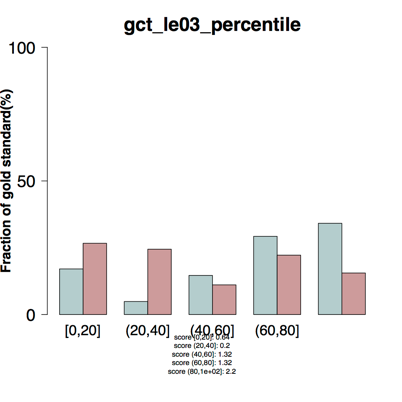

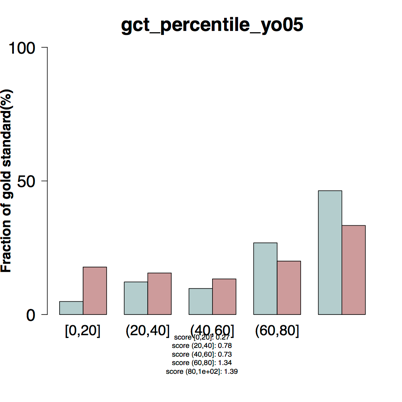


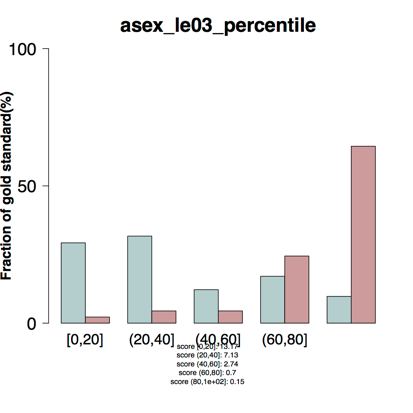

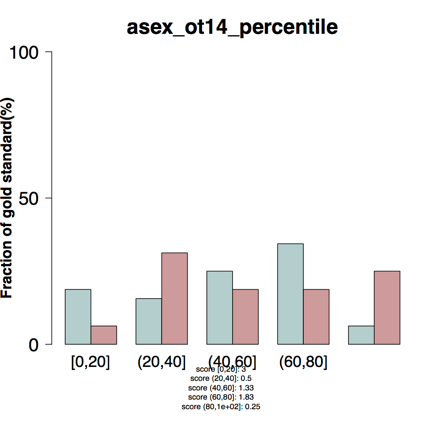

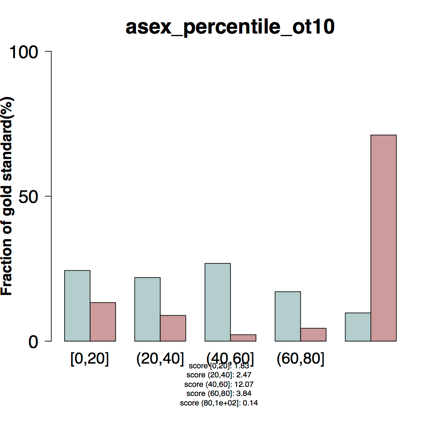

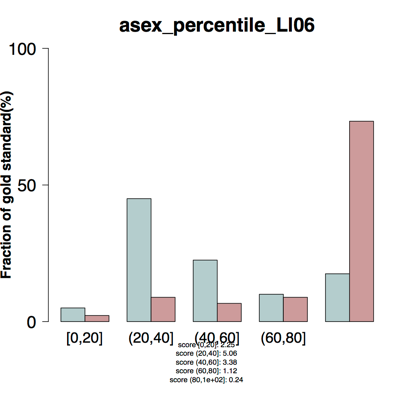


Supplementary Figure 2 (continues on next page)


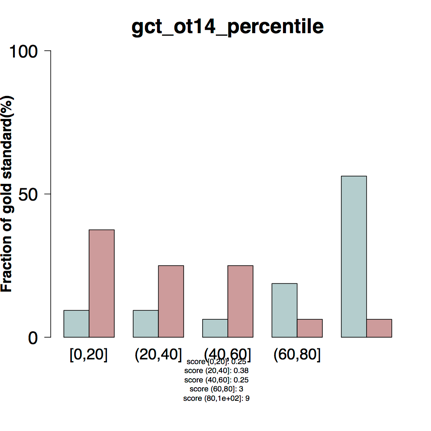

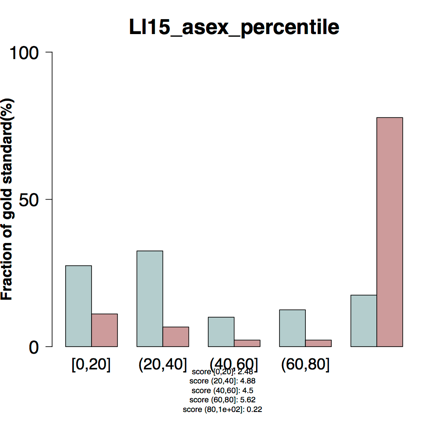


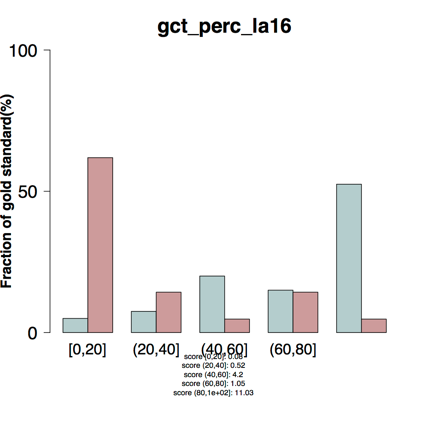


**
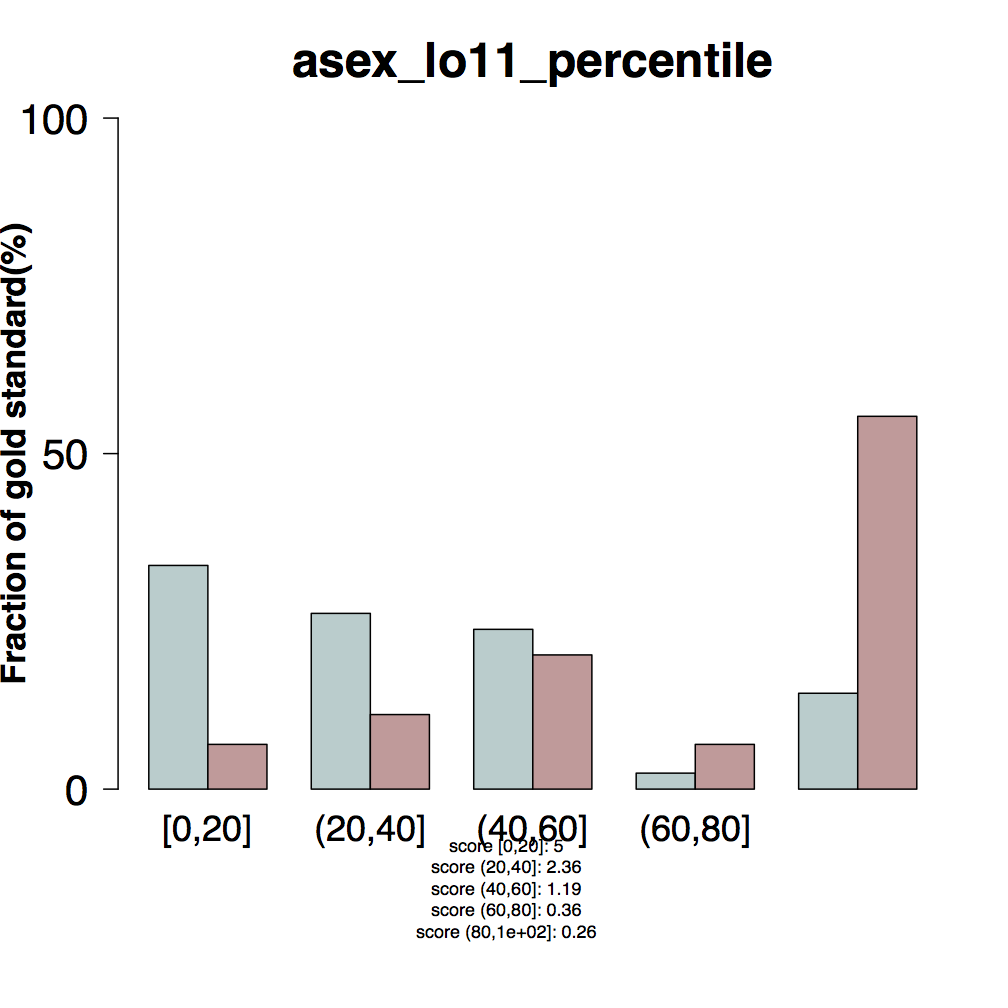
**

**
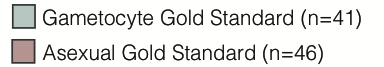

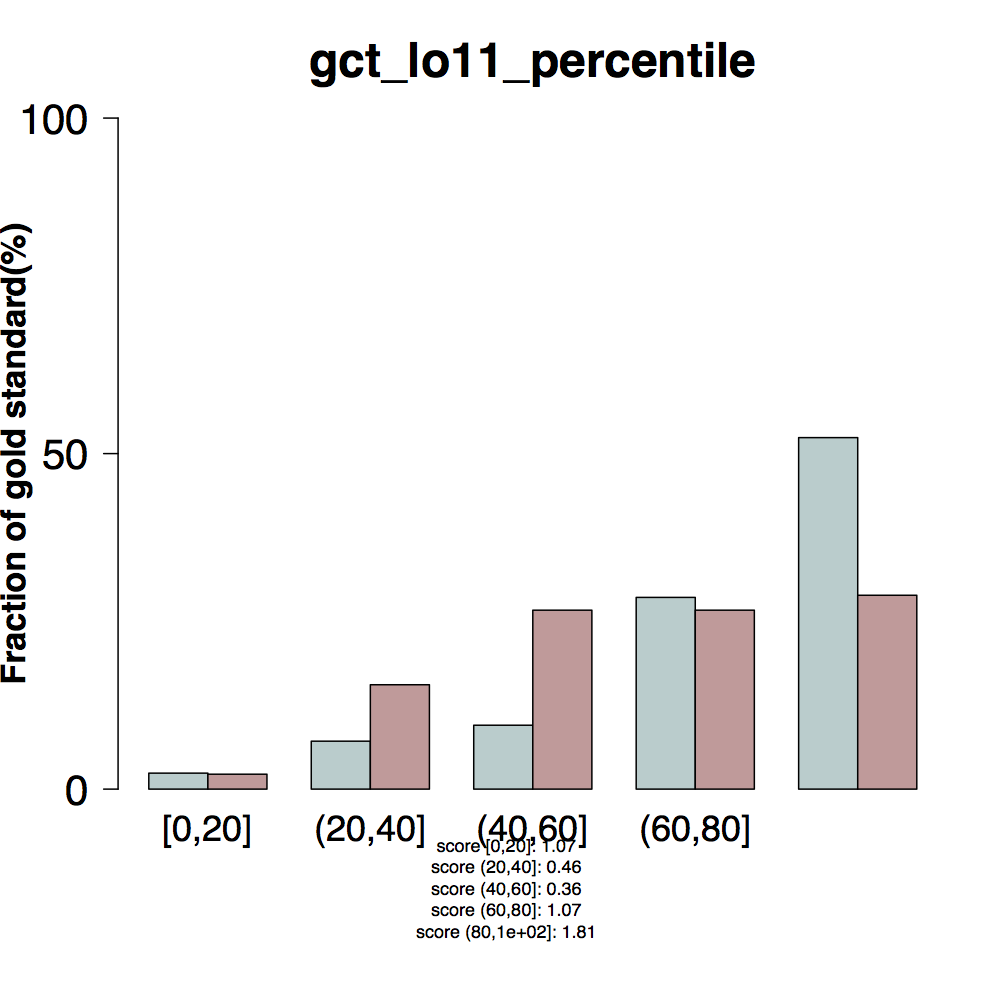
**

**Supplementary figure 2**. Separation of gametocyte (blue) and asexual (red) gold standard (GS) genes by transcriptomics studies. See Table 1 for study keys. Bins for percentiles of the genes ranked for expression. Scores for each bin are calculated as the log2-transformed fraction of gametocyte GS genes over asexual GS genes retrieved in the respective bin.


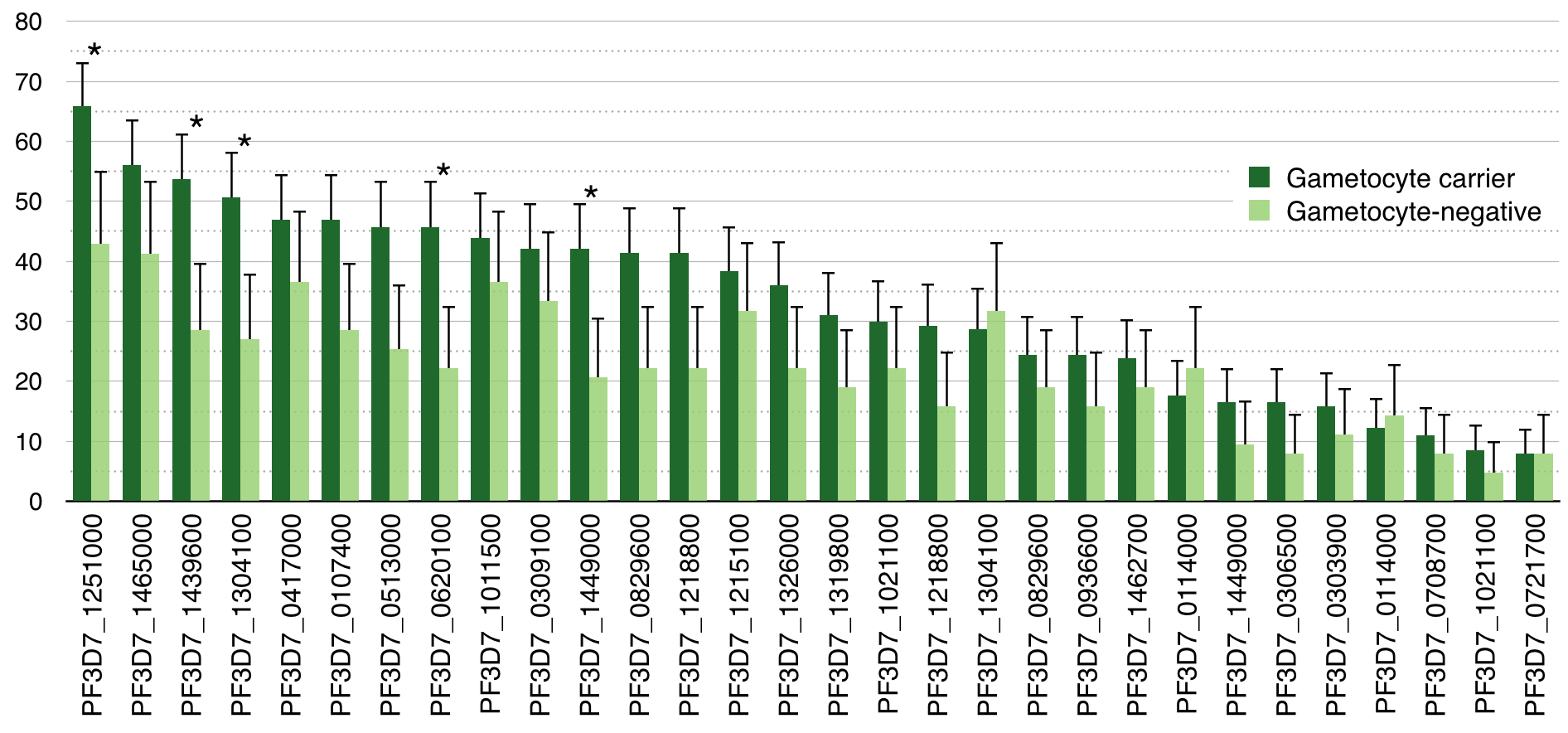


| Seroprevalence (%) |
| --- |

**Supplementary figure 3.** Recognition of gametocyte specific antigens by Gambian parasite carriers that were positive (dark green) or negative (light green) for gametocytes by microscopy. Included are antigens from the top 100 most gametocyte-specific proteins, excluding the gold standard. Error bars show the upper bound of the 95% confidence interval of the proportion. *statistically significant, p<0.05 Fisher’s exact, corrected for multiple testing (Benjamini-Hochberg)


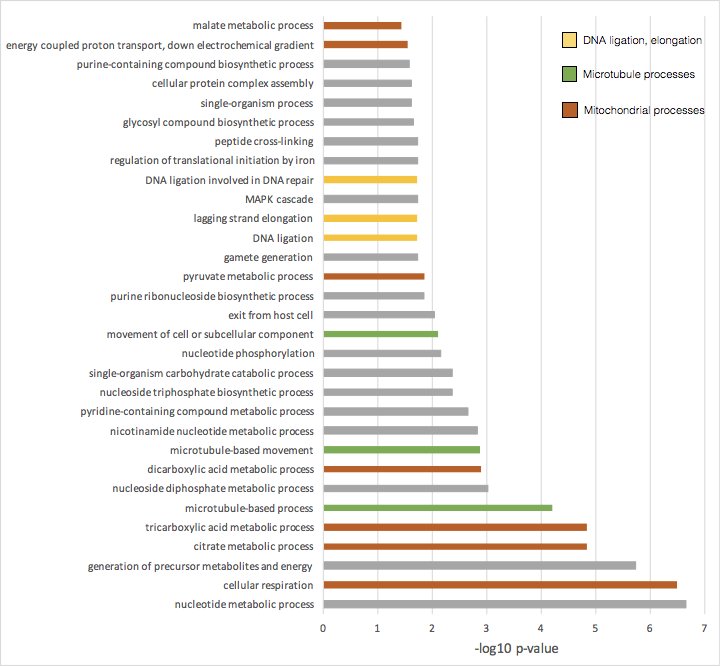


**Supplementary figure 4**. GO term-enrichment of Biological Processes of the 100 highest scoring gametocyte-specific proteins, analyzed with the TopGO package in R. Significant terms (p < 0.05) were checked for semantic redundancy using Revigo. Microtubule-related terms are highlighted in green, DNA ligation and elongation in yellow and mitochondrion-based processes in orange.


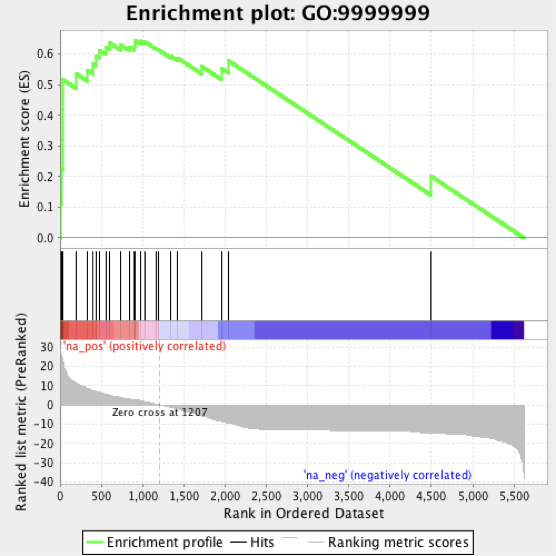


**Supplementary figure 5.** Gene set enrichment analysis of the newly assembled GO term “cilium” for Pf. Genes were ranked according to their gametocyte specificity scoring from proteomics data sets (Supplementary Table 2 and 6).


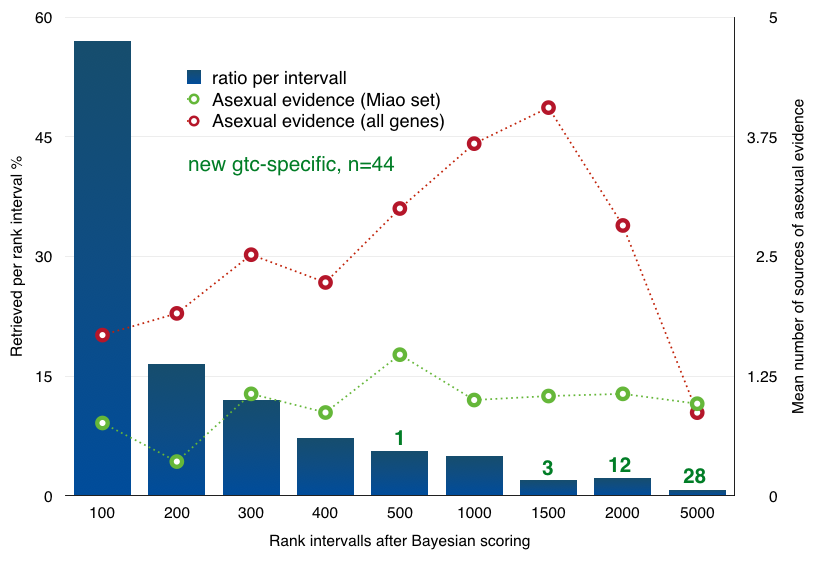
A


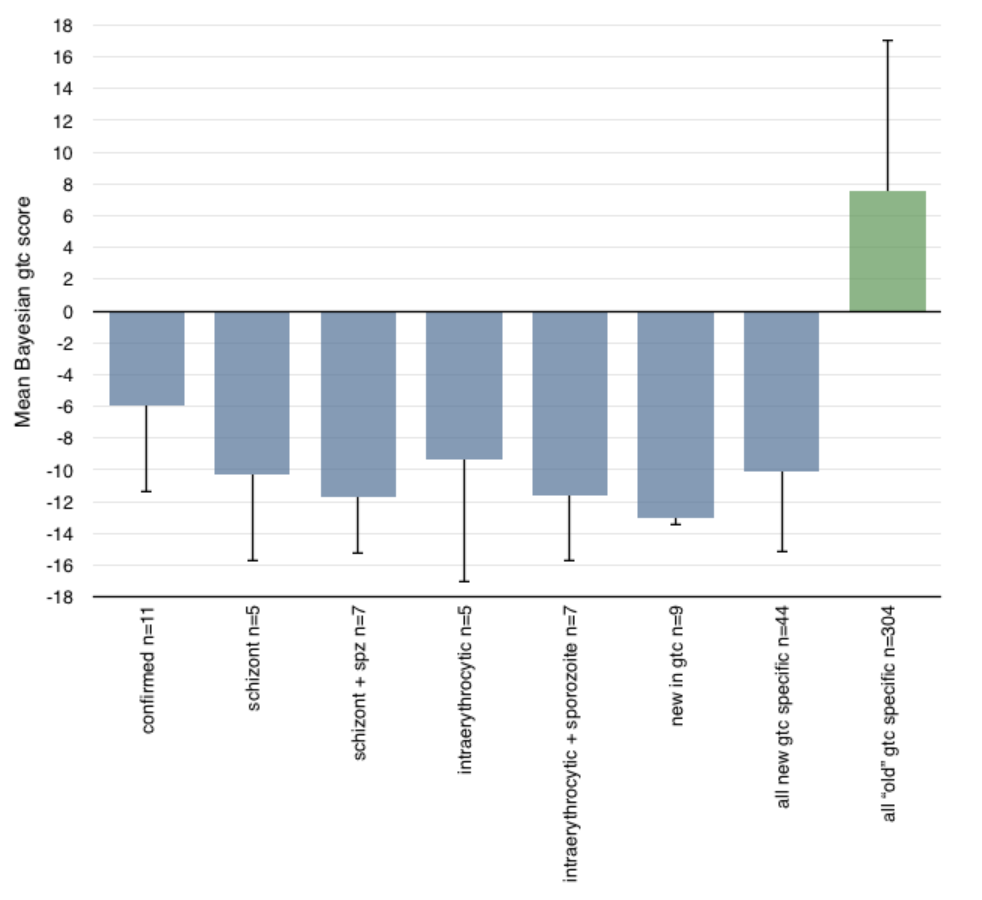
B

**Supplementary figure 6**. Validation of described gametocyte specific proteins by Miao et al. **A**. Previously described (“old”, n=304) gametocyte proteins are mainly found within the top 100 of the Bayesian ranking, newly identified gametocyte proteins (n=44) are ranking lowly due to lack of mass spec evidence. Mean number of asexual sources per protein is plotted in red and green for all *Pf* or all gametocyte proteins (Miao et al.), respectively. **B** Mean Bayesian gametocyte scores for Miao et al.’s new gametocyte proteins and the “old” gametocyte proteins described by Miao et al. The new gametocyte proteins are split into bins according to additional (asexual) mass spec evidence. “confirmed” means a single gametocyte sample with mass spec evidence for the Bayesian analysis.

1. Centre for Molecular and Biomolecular Informatics, Radboud Institute for Molecular Life Sciences, Radboud university medical center, Nijmegen, The Netherlands [↑](#footnote-ref-2)
2. Department of Medical Microbiology, Radboud university medical center, Nijmegen, The Netherlands [↑](#footnote-ref-3)
3. Department of Immunology and Infection, Faculty of Infectious and Tropical Diseases, London School of Hygiene & Tropical Medicine, London, United Kingdom [↑](#footnote-ref-4)
4. Department of Pathogen Molecular Biology, Faculty of Infectious and Tropical Diseases,

   London School of Hygiene & Tropical Medicine, London, United Kingdom [↑](#footnote-ref-5)
5. Dipartimento Malattie Infettive, Istituto Superiore di Sanità, Rome, Italy [↑](#footnote-ref-6)
6. Department of Parasitology, Leiden University Medical Center, Leiden, The Netherlands [↑](#footnote-ref-7)
7. Wellcome Trust Center for Molecular Parasitology, Institute of Infection, Immunity and Inflammation, College of Medical Veterinary & Life Sciences, University of Glasgow, Glasgow, Scotland, United Kingdom

   ^8^ Antigen Discovery Inc., Irvine, California, USA

   ^#a^ Current Address: Centre for Molecular Medicine and Therapeutics, Department of Medical Genetics, BC Children’s Hospital Research Institute, University of British Columbia, Vancouver, BC, Canada

   ^#b^ Current Address: Theoretical Biology and Bioinformatics, Department of Biology, Utrecht University, Utrecht, The Netherlands

   * Corresponding author

   Email Martijn.Huijnen@radboudumc.nl (MAH)

   ^¶^ These authors contributed equally to this work. [↑](#footnote-ref-8)
8. [↑](#footnote-ref-9)
